# Alternative splicing of the *Izumo1* gene ensures triggering gamete fusion in mice

**DOI:** 10.1038/s41598-019-40130-7

**Published:** 2019-02-28

**Authors:** Takako Saito, Ikuo Wada, Naokazu Inoue

**Affiliations:** Department of Cell Science, Institute of Biomedical Sciences, School of Medicine, Fukushima Medical University, 1 Hikarigaoka, Fukushima City, Fukushima, 960-1295 Japan

## Abstract

IZUMO1 is a sperm acrosomal membrane protein that is essential for mammalian fertilization through recognition of JUNO on the oocyte surface and accompanying IZUMO1-JUNO complex formation. Here, we report a new *Izumo1* gene splicing variant (IZUMO1_v2) with a unique 52-amino-acid-long signal sequence transcribed from Exon 1b. Although the mRNA amount of *Izumo1_v2* is 76 times lower than that of the original *Izumo1* (IZUMO1_v1) in the testis, the cell-oocyte assay indicates that IZUMO1_v2-expressing COS-7 cells have the ability to attach to the oocyte equivalent of IZUMO1_v1. To clarify the physiological function of IZUMO1_v2, we produced an IZUMO1_v1-specific knockout mouse line with a nine-base deletion adjacent to the initial methionine codon of IZUMO1_v1 by the CRISPR/Cas9 system. The IZUMO1_v1 knockout male mice carry 0.19-fold lower level of IZUMO1 protein in the spermatozoon; however, reduction in fertility was only minimally affected compared to the wild-type mice, suggesting that only a small fraction of IZUMO1 is sufficient for triggering sperm-egg fusion. We propose that the alternative splicing generating IZUMO1_v2 might function as a fail-safe in mouse for when splicing is disturbed.

## Introduction

In fertilization, two kinds of haploid cells, spermatozoa and oocytes, merge with each other to generate a new individual creature. The exact molecular mechanism underlying the process of fertilization is largely unknown. In particular, the sperm-egg fusion, which is the final step in sexual reproduction, remains to be elucidated. Recently, targeted gene defect studies have reported four essential factors, IZUMO1 and sperm acrosome associated 6 (SPACA6) on the sperm side and IZUMO1-receptor JUNO and cluster of differentiation 9 (CD9) on the ovum side, for triggering gamete fusion^1–6^. In a previous study to clarify how IZUMO1 interacts with JUNO, we determined the tertiary structures of the human IZUMO1-JUNO complex at atomic resolution^7^. Furthermore, we have established an *in vitro* cell-oocyte binding system, in which cultured cells expressing the *Izumo1* gene, such as COS-7 cells, become adhesion-competent towards oocytes^8^. A reconstituted assay revealed that JUNO is excluded from the contact site once it recognizes IZUMO1^9^, which robustly establishes firm adhesion of the two cells^8^. These studies strongly implied that there has to be a secondary receptor for IZUMO1. Since COS-7 cells solely expressing the *Izumo1* gene never acquire membrane fusion activity with oocytes^8^, sperm-egg fusion is considered to consist of multiple steps.

IZUMO1 is a type I transmembrane protein with a large extracellular region, which consists of a helical bundle IZUMO domain^10^ with a conserved cluster of eight cysteines and an *N*-glycosylated immunoglobulin-like domain^7,11–13^, as well as a single transmembrane region with a short cytoplasmic tail. The IZUMO domain includes a central β-hairpin region that provides the main platform for JUNO binding^7,11^. Considering that the *Izumo1* gene has been found to be a haploid-specific expression because *Izumo1* mRNA was detected by reverse transcription polymerase chain reaction (RT-PCR) of the testes only from three-week-old mice^14^, it is believed that IZUMO1 must be a specialized gene that plays a role in gamete recognition and adhesion. Despite the importance of IZUMO1, there have been no detailed reports on *Izumo1* gene regulation leading to appropriate physiological function.

In the current study, we identified a novel transcript of the *Izumo1* gene with a longer signal sequence generated by alternative splicing, and named it IZUMO1 variant 2 (IZUMO1_v2). In order to clarify the function of IZUMO1_v2, we generated an IZUMO1_v1-specific knockout mouse line using the CRISPR/Cas9 system, and investigated the detailed reproductive phenotype *in vitro* and *in vivo*.

## Results

### Validation of a novel *Izumo1* transcript variant 2

**Figure 1:**
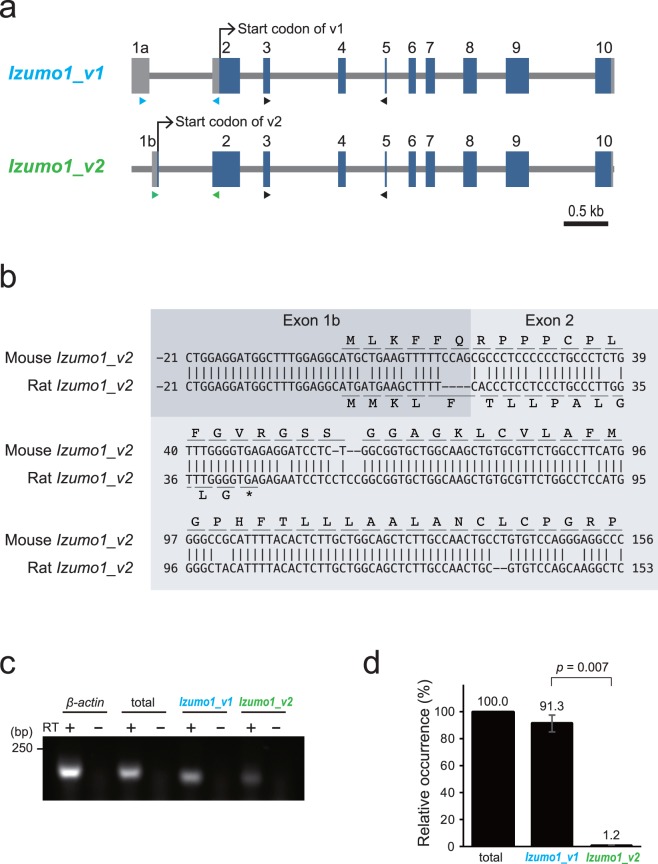
Identification of new mouse *Izumo1* splice variant. (**a**) Diagram of splice variants of mouse *Izumo1* gene. The arrowheads show three specific primer pairs for RT-PCR and RT-qPCR: variant 1 (blue); variant 2 (green); and common to both variants (total: black). (**b**) Alignment of mouse and rat *Izumo1_v2* sequences with their coded amino acid sequences in Exon 1b and 2. Numbering starts from the *Izumo1_v2* start codon. (**c**) RT-PCR for amplification of *Izumo1* variants mRNA from wild-type mouse testis. *β-actin* was used as an internal control. total; amplification of both *Izumo1* variants. Reverse transcriptase (RT)-free samples were used as negative control. (**d**) Relative expression levels of *Izumo1* variants by RT-qPCR analysis in wild-type mouse testis (n = 3). The error bars represent the standard error of three biological replicates (Student’s *t*-test).

We previously identified a mouse *Izumo1* gene encoding a 1,194-nucleotide open reading frame (ORF) (397 amino acids) as a haploid-specific protein (hereafter referred to as “IZUMO1_v1”) (accession number: AB195681)^2,14^. Regarding the alternative splice variants of the *Izumo1* gene, the *Izumo1* transcript variant 2 (*Izumo1_v2*) was predicted in the NCBI database (accession number: XM_006541222.3). *Izumo1_v2* encodes a 1,287-nucleotide ORF (428 amino acids). Indeed, we determined the *Izumo1_v2* sequence from mouse (C57BL/6 strain) testis cDNA by RT-PCR using *Izumo1_v2*-specific primers (accession number: LC426749) (Fig. 1a).

The *Izumo1_v2* transcript consists of ten exons with Exon 1b, which is located 40-bp downstream from Exon 1a of the *Izumo1_v1* (Fig. 1a). Exon 1b has 76 nucleotides with AG, a splicing consensus sequence, at its 3’ end. In addition, Exon 1b includes the start codon of IZUMO1_v2 and is bound to the next common Exon 2 (Fig. 1b). Surprisingly, this *Izumo1_v2* was translated exclusively in mouse (*Mus musculus*) because we could not find the in-frame transcript among other species including other rodents, in the latest NCBI database (Fig. 1b shows an example of rat [Wistar], accession number: LC426750).

To distinguish between the transcripts of *Izumo1_v1* and *v2*, we designed specific primer sets for the amplification of each splice variant (Fig. 1a, blue and green arrowheads, respectively). In addition, one primer pair was designed to amplify both variants to analyse the relative mRNA levels of *Izumo1_v1* and *v2* (Fig. 1a, black arrowhead). RT-PCR experiments using first strand cDNA from mouse testes confirmed that the transcript corresponding to *Izumo1_v2* was present, albeit at very low levels (Fig. 1c). Likewise, more precisely, the RT-quantitative PCR (qPCR) of testis mRNA, in which we employed a previously established mathematical approach for the quantification of splice variants^15^, showed that the *Izumo1_v1* transcript is approximately 76 times more abundant than that of *Izumo1_v2* (Fig. 1d). This indicates that a tiny amount of *Izumo1_v2* definitely exists in mouse testes, although the mutual gene regulation and functional relationship are unknown.

### Expression and biological function of IZUMO1_v2

**Figure 2:**
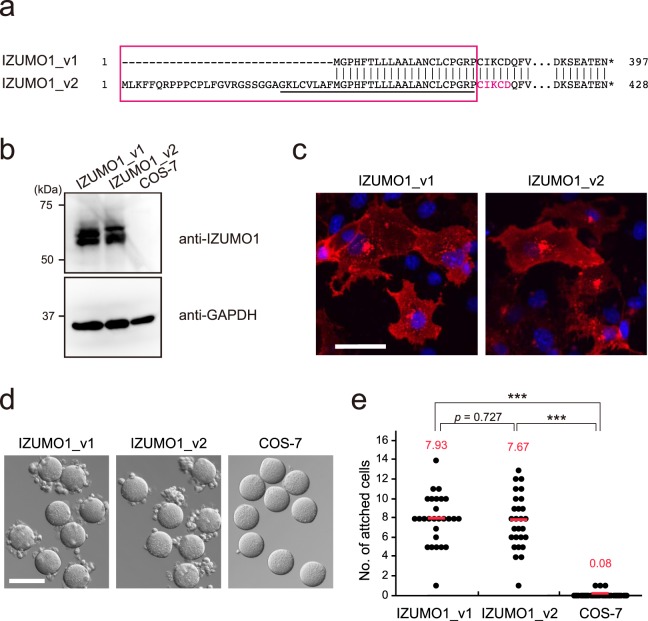
Characterization of IZUMO1_v2. (**a**) Comparison of the amino acid sequences of each variant. The signal sequence (red box) and result of N-terminal sequencing of IZUMO1_v2 (red text) are marked. Underlined residues are predicted C-domain of the NtraC domain architecture. (**b**) Immunoblot analysis of the lysate of IZUMO1_v1 or v2-expressing COS-7 cells and non-transfected COS-7 cells with anti-IZUMO1 (Mab18) and anti-GAPDH (internal control). (**c**) Cell surface localization of IZUMO1_v2. The transfected cells were stained with anti-IZUMO1 antibody (Mab18-Alexa546) and the nuclei were stained with Hoechst 33342. Scale bar, 50 μm. (**d**) Cell-oocyte assay. IZUMO1_v1 or v2-expressing COS-7 cells are attached to mouse oocytes. Scare bar, 100 μm. (**e**) The number of attached cells per oocyte from three independent experiments. The red lines and numbers indicate the average. ***p < 0.001 (Student’s *t*-test).

Compared to IZUMO1_v1, IZUMO1_v2 has a unique 52-amino-acid-long signal sequence, with an additional 31 amino acids (Fig. 2a). The N-terminal amino acid sequencing analysis of the IZUMO1_v2 secretory ectodomain (1Met-350Arg), which was expressed in HEK293T cells, was confirmed to be Cys-Ile-Lys-Cys-Asp (Fig. 2a, red text), implying that the putative 52-amino-acid-long leader sequence was cleaved off by signal peptidase at the same location as that of IZUMO1_v1 (Fig. 2a, red box). Interestingly, the novel 52-amino-acid-long leader sequence shows an NtraC organization, a known feature of long signal peptides of a single-pass integral membrane, in which N- and hydrophobic C-domains (GKLCVLAFMGPHFTLLLAALANCLCPGRP) (Fig. 2a, underline) are separated by a loop-forming transition area^16^. When the immunoblot analysis of the cell lysates from IZUMO1_v1 or v2-expressing COS-7 cells was performed, no significant differences were observed regarding molecular weight or protein amounts between IZUMO1_v1 and v2 (Fig. 2b). Furthermore, as observed with IZUMO1_v1, IZUMO1_v2 was present on the cell surface (Fig. 2c) and also accumulated along the secretory organelles, but not in the mitochondria (Supplementary Fig. 1). These results suggest that at least a part of IZUMO1_v2 is correctly folded and transported to the plasma membrane as IZUMO1_v1.

To ascertain the biological function of IZUMO1_v2, we performed a previously described cell-oocyte assay to study sperm-egg recognition and adhesion utilizing cultured cells, such as COS-7 cells, instead of mature spermatozoa^8^. The cell-oocyte assay clearly demonstrated that IZUMO1_v2-expressing COS-7 cells have the ability to attach to the oocyte equivalent to IZUMO1_v1 (Fig. 2d,e). This data suggests that the biological function of IZUMO1_v2 is comparable to that of IZUMO1_v1.

### Analysis of IZUMO1_v1 KO mouse

**Figure 3:**
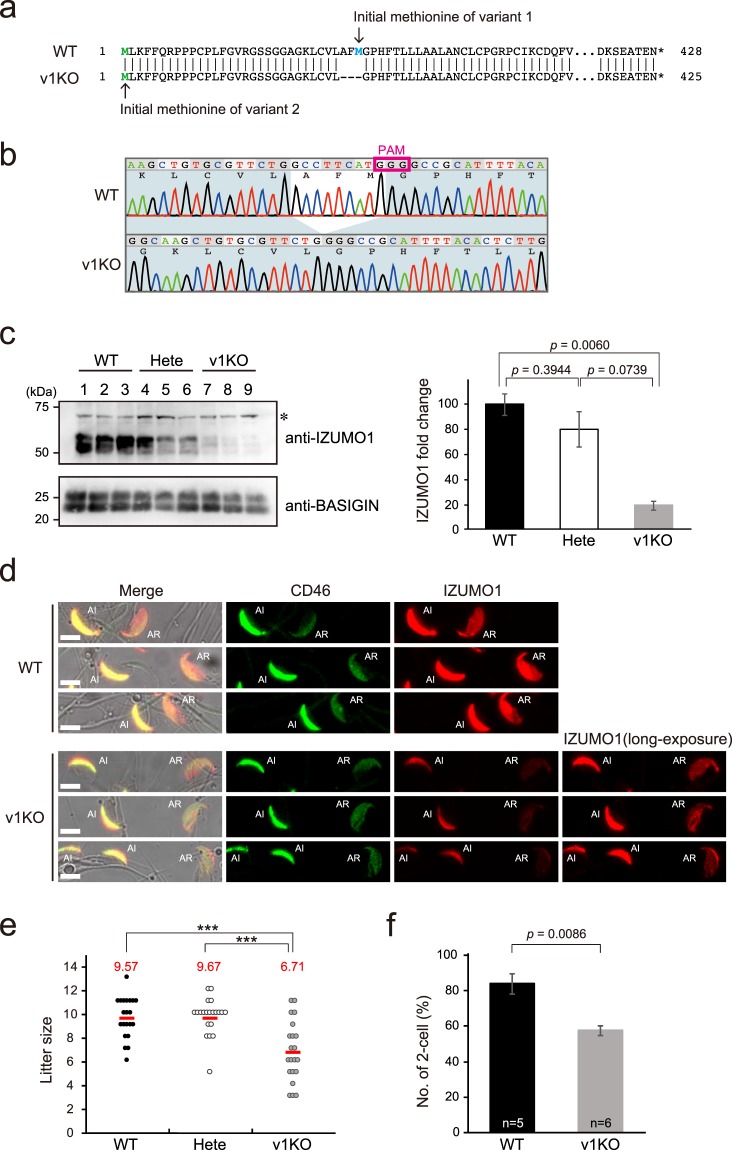
Generation and reproductive analysis of IZUMO1_v1 KO mouse. (**a**) Amino acid sequence of mouse IZUMO1 in wild-type (WT) and IZUMO1_v1 KO (v1KO). The initial methionine of variants 1 and 2 are blue and green, respectively. (**b**) The results of the sequence analysis of the v1KO mouse *Izumo1* gene. The v1KO mouse has a nine-base deletion adjacent to the initial IZUMO1_v1 methionine codon. PAM stands for protospacer adjacent motif. (**c**) Immunoblot analysis of sperm extracts. Each lane represents a different individual of wild-type (WT), heterozygous (Hete) and IZUMO1_v1 KO (v1KO) mouse. BASIGIN was used as a control. The asterisk indicates non-specific band. The average IZUMO1 protein levels of each genotypes are shown as fold-change relative to WT after normalization using the BASIGIN protein level. n = 3. Error bars are SEM. (**d**) Immunocytochemistry of WT and v1KO sperm. Sperm heads were immunostained with anti-CD46 (green) and anti-IZUMO1 antibody (red). The localization patterns of CD46 indicate acrosome intact (AI) and acrosome reacted (AR) sperm. Scale bar, 5 μm. (**e**) Litter size of mating crossed with B6D2F1 females and WT, Hete, and v1KO males. The red lines and the numbers indicate the average. Six different mice for each genotype were used. ***p < 0.001 (Student’s *t*-test) (**f**) *In vitro* fertilization assay using WT or v1KO males (n = 5 and 6, respectively). The fertility of each group is indicated by the average percentage of oocytes that developed to the two-cell stage (Total number of eggs in WT and v1KO are 236 and 283, respectively). Error bars are SEM.

From the above results, we predicted that the IZUMO1 protein on the sperm surface, derived from *Izumo1_v2* mRNA, is also functional for fertilization. To elucidate how IZUMO1_v2 contributes to reproduction, we produced IZUMO1_v1-specific knockout mice (IZUMO1_v1 KO) utilizing the CRISPR/Cas9 system. To minimize the influence of genomic modification, we designed a single guide RNA (sgRNA) to target the start codon of the *Izumo1_v1* gene (Fig. 3a), and injected pX330-sgRNA plasmids into the pronuclei of fertilized eggs^17^. As a result, we were able to obtain the IZUMO1_v1 KO mouse carrying a deletion mutant of IZUMO1_v1 (p.Met1del) / IZUMO1_v2 (p.Ala30-Met32del) (Fig. 3b).

This mouse line is supposed to produce the IZUMO1 protein from the transcript of *Izumo1_v2* alone due to the deletion of IZUMO1_v1’s initial methionine (Fig. 3a,b). Indeed, immunoblot analysis demonstrated that the anti-IZUMO1 antibody detected IZUMO1 proteins in sperm lysates of IZUMO1_v1 KO mice; however, the band signals were very weak compared to those of the wild-type mice (Fig. 3c). The relative protein level of IZUMO1 on the spermatozoon was 0.19-fold lower in the IZUMO1_v1 KO mice than the wild-type mice (Fig. 3c). To validate the predicted amino acid sequence of the IZUMO1_v2 protein, we isolated IZUMO1_v2 from an IZUMO1_v1 KO sperm lysate using an anti-IZUMO1 antibody, and determined the sequence by liquid chromatography tandem mass spectrometry (LC-MS/MS). As a result, all predicted peptides derived from IZUMO1_v2 were identified (Supplementary Fig. 2a,b). Consistent with previously reported results of IZUMO1 KO mice^2^, the spermatozoa of IZUMO1_v1 KO in the current study showed normal morphology and motility. We used CD46, which has similar dynamics to IZUMO1, translocating from the acrosomal cap region to the whole sperm head during the acrosome reaction (AR), as a marker to distinguish between acrosome intact (AI) and AR spermatozoa^18,19^. As we expected, before AR, IZUMO1_v2 was located in the acrosomal cap region, and spread to cover the whole sperm head after AR just as an intrinsic IZUMO1^2,20,21^; however, the IZUMO1 fluorescent intensity was much dimmer than that of the wild-type mice (Fig. 3d).

Next, to evaluate fertility, wild-type, heterozygous and IZUMO1_v1 KO male mice were mated with wild-type females. Intriguingly, the IZUMO1_v1 KO males were still fertile, although their litter sizes were significantly decreased compared to those of the wild-type and heterozygous male mice (Fig. 3e). *In vitro* fertilization (IVF) experiments showed that the fertilization rate (percentage of two-cell embryo development) of the IZUMO1_v1 KO males was significantly lower than that of the wild-type mice (Fig. 3f). Since deletion of the *Izumo1* gene causes complete sterility, these analyses indicate that IZUMO1_v2 can rescue loss of IZUMO1_v1, and, at the same time, suggest that the quantity of mature IZUMO1 proteins is critical for mouse fertility.

## Discussion

Alternative splicing is an important posttranscriptional regulatory mechanism that generates multiple mRNA variants. More than 90% of human genes undergo alternative splicing^22,23^. This process can alter the localization, stability and biological function of the protein by changing the representation of gene isoform^24^. For example, it is known that the splicing variants of reproduction-related genes play important roles in various physiological processes. An angiotensin converting enzyme (ACE) is a well-known regulator of cardiovascular homeostasis in somatic cells. However, the splicing variant of testicular ACE (tACE), transcribed from the testis-specific promoter in the 12th intron of the somatic ACE, functions in male fertility. tACE knockout mice are infertile along with failure of migration into the oviduct and loss of ability to bind to the zona pellucida of eggs^25,26^. The plasma membrane Ca^2+^-ATPase isoform 4 (PMCA4) also has two splice variants, PMCA4a and 4b, exclusively expressed in mouse testes and epididymides. These variants contain a distinct C-terminus domain and display different kinetic properties for Ca^2+^ transport^27^. Since PMCA4 knockout mice fail to achieve the hyperactivated state of sperm motility, both *Pmca4* splice variants are likely required to regulate Ca^2+^ handling in spermatozoon, and are crucial for male fertility^28,29^.

IZUMO1 is essential for gamete fusion. Although the spermatozoa of IZUMO1 knockout mice have normal morphology and physiology, these mice are sterile because their spermatozoa lack the ability to fuse with oocytes^2^. Despite the apparent importance of IZUMO1 in reproduction, as far as we know, no studies on transcriptional regulation have been reported. We described here a novel *Izumo1* transcript, named IZUMO1_v2, that has a unique 52-amino-acid signal sequence, produced by alternative splicing transcribed from Exon 1b. Organization of the long signal sequence fully meets the requirements of the NtraC model for a single-pass integral membrane protein^16^. It should be noted that IZUMO1_v2 is different from a typical NtraC-organized long signal sequence; the short N-domain appears to lack an obvious targeting signal for other organelles such as mitochondria, and the C-domain alone should be sufficient for targeting/translocation of the endoplasmic reticulum membranes because the signal sequence of IZUMO1_v1 is included in the C-domain. Indeed, the results of our analyses indicate that IZUMO1_v2-expressing COS-7 has no significant differences in protein expression, localization or function between IZUMO_v1 and v2 (Fig. 2). Evidently, the long leader sequence of IZUMO_v2 does not affect protein stability. This is likely because the signal sequence is co-translationally removed and should not affect the posttranslational events. In contrast, the cytoplasmic tailless IZUMO1 causes mRNA degradation and protein instability, thereby the mice harbouring this mutation showed a 0.33-fold reduction in IZUMO1 protein expression in spermatozoa despite normal fertility^18^.

Though not a major transcript of the IZUMO1 protein, it is likely that *Izumo1_v2* can serve as a fail-safe for when the *Izumo1_v1* transcript is not produced for any reason, because 1) this gene product has the same mature protein sequence, 2) it exhibited comparable function as IZUMO1_v1 *in vitro*, and 3) its expression was sufficient for fertility. This may relate to the mechanism of IZUMO1 mediated membrane fusion. Although we previously reported that JUNO is lost from the contact site after IZUMO1-JUNO recognition^9^, the exact mechanism on how fusion is initiated in paring two membranes is elusive. We now speculate that a limited amount of IZUMO1 protein may trigger a cascade reaction to disturb the lipid bilayer of the oocyte, leading to fusion of the two cell membranes. Because IZUMO1 molecules are concentrated on the fusion site of sperm plasma membranes (equatorial segment) after the AR, a minimal amount of IZUMO1 may be sufficient for further reactions.

Intriguingly, a phylogenetic analysis (FigTree v1.4.3 using CLUSTALW) showed that the Exon 1b of IZUMO1_v2 is conserved among some rodents (Ryukyu mouse, shrew mouse and rat) that are evolutionally very close to the house mouse. However, the predicted transcript prematurely terminates and the ORF is not functional. We believe that IZUMO1_v2 is evolved in mouse to compensate for the vulnerability of the fertilization process. The proteins involved in reproduction have evolved rapidly. For instance, a disintegrin and metalloprotease 3 (ADAM3) plays crucial roles in male fertility in mice; however, they are no longer used in human (there is as a pseudogene)^30^. A recent expansion of NCBI database has indicated that IZUMO1 orthologues are well conserved among not only mammals but also other vertebrates (bony fish, amphibians and reptiles etc.). On the other hand, IZUMO1’s counterpart receptor JUNO is only conserved among mammals, suggesting mammalian specific neofunctionalization of the ancestral folate receptors and their coevolution within mammals due to heterogeneous evolutionary forces^31^.

In the present study, unexpected findings emerged through the reproductive analysis of IZUMO1_v1 specific knockout mice. Thus, we believe that further studies using genetically modified animals are needed to clarify the precise molecular mechanism of fertilization. This will benefit the clinical treatment of sterility and support the potential development of novel contraceptive strategies in the near future.

## Methods

### Mice

B6D2F1 and ICR mice were purchased from Japan SLC Inc. All animal studies were approved by the Animal Experiments Committee of Fukushima Medical University, Japan, and performed under the guidelines and regulations of Fukushima Medical University.

### RT-PCR and Quantitative real time PCR (RT-qPCR)

Total RNA was extracted from a single testis of a wild-type mouse using NucleoSpin RNA Plus (MACHEREY-NAGEL) and treated with rDNase (MACHEREY-NAGEL). First-strand cDNA synthesis was performed by PrimeScriptII 1st strand cDNA Synthesis Kit (Takara Bio). The following three primer pairs were designed to amplify variants 1 and 2, as well as the common region shared by both variants (total). v1, forward (5′-GAGCTGGGTGAAGCATTAGC-3′); v2, forward (5′-GCCTTGAGGAAGAGTAGACAGG-3′); v1 and v2, reverse (5′-TCCTCTCACCCCAAACAGAG-3′); total, forward (5′-CAGACAGTGACTTAAAAGGAGAGC-3′); reverse (5′-GCATTTGTTGGGACAAAGAAC-3′). *β-actin* was employed as a reference gene using a forward (5′-TGACAGGATGCAGAAGGAGA-3′) and reverse (5′-GCTGGAAGGTGGACAGTGAG-3′) primer set^32^. RT-PCR was carried out using Ex Taq (Takara bio), and quantitative PCR was performed using TB Green Premix Ex Taq (Takara bio) and LightCycler (Roche). Calculation of the relative incidence of the spliced transcripts was performed as previously described^15^.

### Cloning of *Izumo1_v2*

The primer sequences used for cloning mouse *Izumo1_v2* were forward (5′-GCCTTGAGGAAGAGTAGACAGG-3′) and reverse (5′-TAAGTCAAATACAAATCTT-3′), and those used for rat *Izumo1_v2* were forward (5′-AAGTAAACAGGTGCCCCAGA-3′) and reverse (5′-ACTGAGACAACACATCAAATCTTTA-3′). PCR was performed with KOD-Plus-Neo (Toyobo) and first-strand cDNA from mouse (C57BL/6) or rat (Wistar) testis (Genostaff). PCR products were ligated into the pCR II-Blunt-TOPO vector (Thermo Fisher Scientific) and the plasmids from randomly selected positive colonies were sequenced. The accession numbers for the mouse and rat *Izumo1_v2* sequences are LC426749 and LC426750, respectively.

### Cell culture and transient transfection

COS-7 and HEK293T cells were grown in Dulbecco’s modified Eagle’s medium (high glucose) (Nakalai Tesque) with 10% (v/v) fetal bovine serum (Sigma-Aldrich), 1 mM sodium pyruvate, 1 × non-essential amino acids, 2 mM L-Glutamine, 1,000 U/L penicillin and 1 mg/L streptomycin (Thermo Fisher Scientific) at 37 °C with 5% CO_2_. For expression of transmembrane or the secretory form of the IZUMO1 proteins, cDNA fragments including PA tag (GVAMPGAEDDVV) or Twin-Strep-tag (SAWSHPQFEKGGGSGGGSGGSAWSHPQFEK), were cloned into mammalian expression vector pCXN-2^9^, respectively. Plasmids were prepared using NucleoBond Xtra Midi (MACHEREY-NAGEL). Transient transfections were performed as described previously^33^, and cells were cultured in serum free medium.

### Immunoblotting of transfected cells

After 72 hours’ transfection, the COS-7 cells were washed three times using PBS, and removed from their cultured plates using 5 mM EDTA-PBS at 37 °C for 30 min. The cells were then collected using centrifugation at 5,000 × g for 5 min, and treated with 1% Triton-X100 and protease inhibitor cocktail (FUJIFILM Wako Pure Chemical Corporation) in PBS for 1 hour on ice. After centrifugation at 17,000 × g for 5 min, whole-cell lysates from IZUMO1_v1 or IZUMO1_v2 transfected COS-7 and non-transfected COS-7 cells were used for immunoblotting. The membranes were probed with primary antibodies (anti-IZUMO1 [Mab18]^9^ and anti-GAPDH [FUJIFIM Wako Pure Chemical Corporation]) followed by secondary antibodies conjugated to HRP (Jackson ImmunoResearch Laboratories). Chemiluminescence reactions were performed with ECL Prime (GE Healthcare Life Sciences).

### Fluorescence imaging for COS-7 cells

Regarding live cell surface staining, IZUMO1_v1 or v2-expressing COS-7 cells on the glass-bottom dish (MatTek Corporation) were washed with PBS, and incubated with 0.5 μg/ml anti-IZUMO1 antibody (Mab18-Alexa546) and 1 μg/ml Hoechst 33342 (Sigma-Aldrich) for 2 hours at 37 °C. As for cytoplasmic staining, IZUMO1_v1 or v2-expressing COS-7 cells were incubated with 500 nM MitoTracker Red CMXRos (Thermo Fisher Scientific) at 37 °C for 30 min and fixed in 1% paraformaldehyde/PBS for 1 hour, followed by incubation with 0.2% Triton-X100/PBS for 10 min. After washing three times with PBS, fixed cells were covered with blocking buffer (1% BSA in PBS) for 30 min and incubated with 0.5 μg/ml anti-IZUMO1 antibody (Mab18-Alexa488) and 1 μg/ml Hoechst 33342 for 1 hour at 37 °C. After labelling, the cells were washed and observed under an A1R confocal microscope (Nikon) with a 40 × objective (NA 0.95).

### Cell-oocyte assay

Forty-eight hours after transfection, the COS-7 cells were treated with 5 mM EDTA-PBS at 37 °C for 10 min, washed three times with PBS by centrifugation at 2,000 × g for 1 min, and suspended in TYH medium (LSI Medience)^34^. The zona pellucida of eggs were dissolved by 1.0 mg/ml of collagenase (FUJIFILM Wako Pure Chemical Corporation) and zona-free oocytes were incubated with the collected COS-7 cells at 37 °C in the TYH medium for 1 hour. The oocytes were then washed and moved to drops of TYH medium, and the attached cells were counted under an inverted microscope.

### Generation of IZUMO1_v1 KO mice

A pair of oligonucleotides (5′-CACCCTGTGCGTTCTGGCCTTCAT-3′ and 5′-AAACATGAAGGCCAGAACGCACAG-3′) as a guide sequence were annealed and cloned into a *Bbs*I cut pX330 vector (Addgene). pX330 plasmid DNA was purified and diluted into 5 mM Tris-HCl pH 7.4/0.1 mM EDTA of a final 5 ng/μl concentration^17^. Plasmid DNA was injected into the pronuclei of fertilized eggs with glass capillaries attached to a micromanipulator (FemtoJet, Eppendorf). After microinjection, the zygotes were transferred and cultured in KSOM (ark-resource)^35^ at 37 °C with 5% CO_2_, and two-cell embryos were transferred into pseudopregnant ICR female mice. F3 and F4 generation mice were used for the experiments and all genotyping was performed by PCR on tail-tip DNA using 5′-GTGCGTTCTGGCCTTCATGG-3′ for wild-type or 5′-AAGCTGTGCGTTCTGGGGCC-3′ for IZUMO1_v1 KO and 5′-ACTGTCTAGCAAGATTGTTAAAGATGTCC-3′ as a forward and a reverse primer, respectively. IZUMO1_v1 KO mice were deposited at the RIKEN BioResource Centre (accession number: RBRC10184.)

### Immunoblotting analysis of spermatozoa

Spermatozoa were collected from the cauda epididymis and vas deference of >12-week-old male mice. The spermatozoon proteins were solubilized in PBS with 1% Triton-X100 and protease inhibitor cocktail (FUJIFIM Wako Pure Chemical Corporation) for 1 hour on ice. After centrifuge at 17,000 × g for 30 min, the supernatant protein concentration was determined. Then, 20 μg of sperm proteins were separated using SDS-PAGE, transferred to a PVDF membrane (Millipore), and probed with anti-IZUMO1 antibody (Mab18)^9^ or anti-BASIGIN antibody (sc-9757: Santa Cruz Biotechnology).

### Immunoprecipitation and LC-MS/MS analyses

For immunoprecipitation, sperm extract from five IZUMO1_v1 KO male mice was incubated with 10 μg of anti-IZUMO1 monoclonal antibody (Mab34) overnight at 4 °C, followed by incubation with 15 μl of anti-rat IgG conjugated Sepharose 4B beads (Thermo Fisher Scientific) for 3 hours at 4 °C. The beads were washed three times in 1% Triton-X100, 50 mM Tris-HCl (pH 8.0), 150 mM NaCl. The proteins were eluted with SDS sample buffer and heated at 100 °C for 5 min. The eluted samples were separated by SDS-PAGE under a non-reducing condition, then subjected to silver staining. 55–60 kDa bands were cut out from the silver-stained gel and subjected by LC-MS/MS analysis.

### Fluorescence imaging for spermatozoa

The spermatozoa were collected from cauda epididymis and incubated in a drop of TYH medium overlaid with mineral oil at 37 °C with 5% CO_2_ for 2 hours. After incubation, the cells were washed with PBS and centrifuged at 900 × g for 5 min, air dried on the slide glasses at 42 °C, fixed with 4% paraformaldehyde in PBS for 5 min at room temperature, and washed three times with PBS. The slide glasses were blocked for 1 hour with 0.1% Tween / 5% BSA in PBS at room temperature, and incubated in the dark with 0.5 μg/ml anti-IZUMO1 antibody (Mab34-Alexa546)^8^ and 1.5 μg/ml anti-CD46-Alexa488^18^ for 2 hours at room temperature. After washing with PBS, the spermatozoa were observed under an A1 confocal microscope (Nikon) with a 60 × objective (NA 1.27).

### *In vitro* fertilization

Mouse spermatozoa were collected from the cauda epididymis and were capacitated *in vitro* for 2 hours in a 200-µL drop of TYH medium that was covered with mineral oil. Superovulation was induced in B6D2F1 female mice (>8 weeks old) by injecting 7.5 IU of equine chorionic gonadotropin (eCG) and 7.5 IU of human chorionic gonadotropin (hCG) at 48-hour intervals. Sixteen hours after hCG injection, oocytes were collected from the oviduct, placed in 100 μl of TYH medium, and incubated with 2 × 10^5^ spermatozoa ml^−1^ at 37 °C with 5% CO_2_. After 4 hours’ insemination, oocytes were washed with TYH medium and incubated until they developed to the two-cell stage.

## Supplementary information

Supplementary Information

## Data Availability

All data of this study are available from the corresponding author (N.I.) on request.
