# Supplementary material for: Alternative splicing of the Izumo1 gene ensures triggering gamete fusion in mice
Source: Sci Rep. 2019 Feb 28;9:3151. doi: 10.1038/s41598-019-40130-7 (PMC6395798; doi:10.1038/s41598-019-40130-7)
Supplement: Supplementary file 1 — Supplementary Information [file 41598_2019_40130_MOESM1_ESM.pdf]

**Supplementary Information**

# Alternative splicing of the *Izumo1* gene ensures triggering gamete fusion in mouse

Takako Saito[1], Ikuo Wada[1] and Naokazu Inoue[1*]

[1]Department of Cell Science, Institute of Biomedical Sciences, School of Medicine, Fukushima Medical University, 1 Hikarigaoka, Fukushima City, Fukushima 960-1295, Japan.

[*]Author for correspondence (n-inoue@fmu.ac.jp)

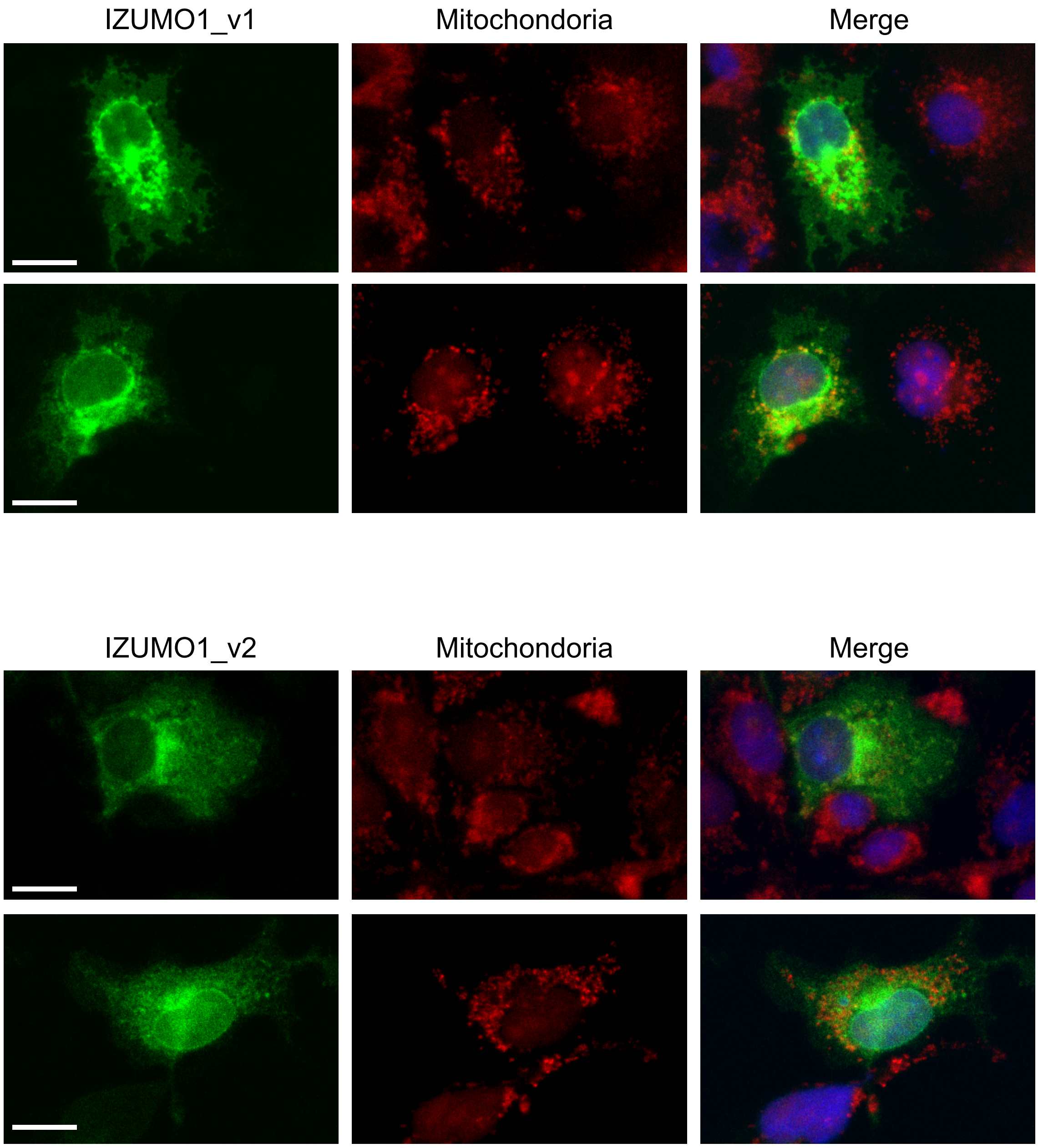


**Supplementary Figure 1. Intracellular localization of IZUMO1_v2.**

IZUMO1_v1 or v2 expressing COS-7 cells were stained with an anti-IZUMO1 antibody (Mab18-Alexa488: green), MitoTracker Red CMXRos (mitochondria: red) and Hoechst 33342 (nuclei: blue) after permeabilization with Triton-X100. Scare bar, 20 μm.

a

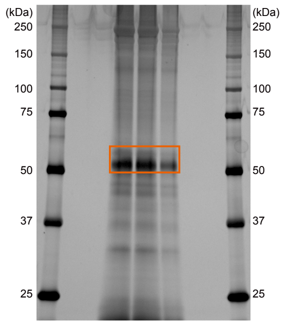


b

```
  1  MLKFFQRPPPCPLFGVRGSSGGAGKLCVLAFMGPHFTLLLAALANCLCPG   50
 51  RPCIKCDQFVTDALKTFENTYLNDHLPHDIHKNVMRMVNHEVSSFGVVTS  100
101  AEDSYLGAVDENTLEQATWSFLKDLKRITDSDLKGELFIKELLWMLRHQK  150
151  DIFNNLARQFQKEVLCPNKCGVMSQTLIWCLKCEKQLHICRKSLDCGERH  200
201  IEVHRSEDLVLDCLLSWHRASKGLTDYSFYRVWENSSETLIAKGKEPYLT  250
251  KSMVGPEDAGNYRCVLDTINQGHATVIRYDVTVLPPKHSEENQPPNIITQ  300
301  EEHETPVHVTPQTPPGQEPESELYPELHPELYPELIPTVAQNPEKKMKTR  350
351  LLILLTLGFVVLVASIIISVLHFRKVSAKLKNASDEVKPTASGSKSDQSL  400
401  SQQMGLKKASQADFNSDYSGDKSEATEN*                       428
```

**Supplementary Figure 2. Determination of amino acid sequence of IZUMO1_v2 by LC-MS/MS analysis.**

(a) Silver-stained gel image of the purified IZUMO1_v2 protein from the IZUMO1_v1 KO sperm lysate. The box indicates the 55–60 kDa bands that were used for the LC-MS/MS analysis.

(b) Full-length amino acid sequence of IZUMO1_v2. Detected peptides from the 55–60 kDa bands are shown in orange.

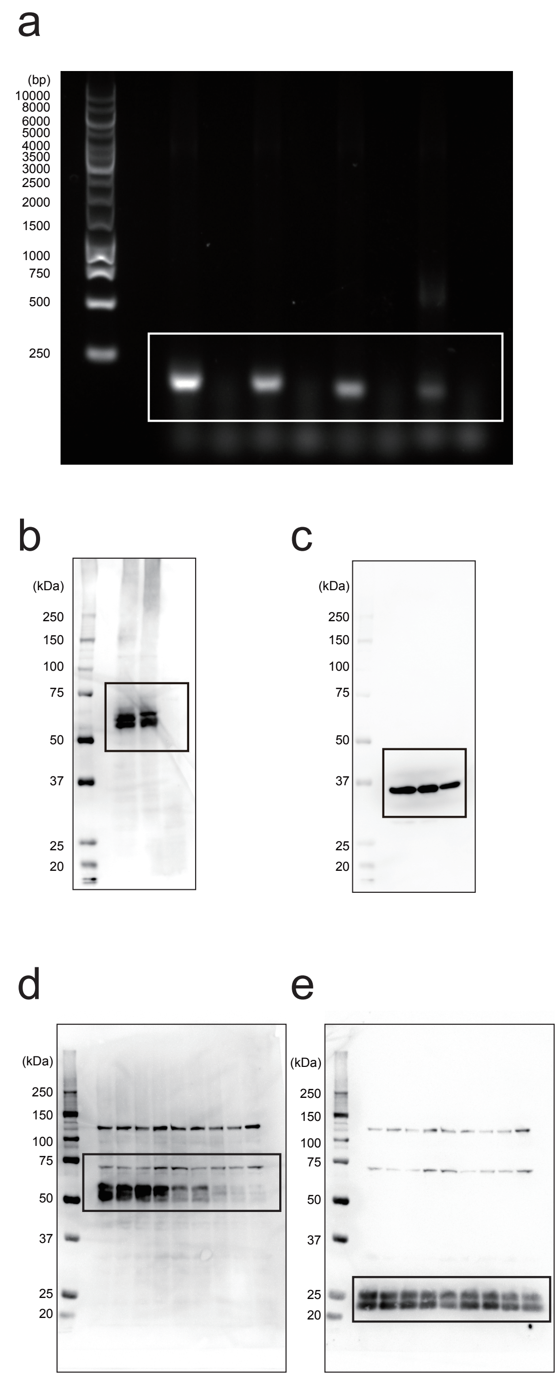


**Supplementary Figure 3. Uncropped full-length gel images.**

(a) Full-length gel of Figure 1c: RT-PCR

(b) Full-length blot of Figure 2b: immunoblot (anti-IZUMO1)

(c) Full-length blot of Figure 2b: immunoblot (anti-GAPDH)

(d) Full-length blot of Figure 3c: immunoblot (anti-IZUMO1)

(e) Full-length blot of Figure 3c: immunoblot (anti-BASIGIN)
